# Successful replantation of total scalp amputation due to dog bites in geriatric patient with prolonged tissue ischemia and comorbidities: a case report

**DOI:** 10.1093/jscr/rjad170

**Published:** 2023-04-12

**Authors:** Mufida Muzakkie, Hilda Nadhila Hasbi, Muhammad Tafdhil Tardha

**Affiliations:** Plastic Reconstructive and Aesthetic Surgery Division, Surgery Department - Mohammad Hoesin Central General Hospital, Faculty of Medicine - Sriwijaya University, Palembang, Indonesia; Plastic Reconstructive and Aesthetic Surgery Division, Surgery Department - Mohammad Hoesin Central General Hospital, Faculty of Medicine - Sriwijaya University, Palembang, Indonesia; Plastic Reconstructive and Aesthetic Surgery Division, Surgery Department - Mohammad Hoesin Central General Hospital, Faculty of Medicine - Sriwijaya University, Palembang, Indonesia

## Abstract

Traumatic scalp amputation is a rare and life-threatening case causing massive blood loss and serious tissue damage. This report aims to record a successful scalp replantation on a 69-year-old woman with total scalp amputation due to dog bites. The challenges of this case were age, prolonged tissue ischemia and several comorbidities such as high glucose index (uncontrolled diabetes mellitus), hypertension and obesity. After 2 hours of admission, 9 hours of stabilization and 7 hours of revascularization, left superficial temporal artery and vein end-to-end anastomosis was successful, adding to 18 hours total of warm tissue ischemia time. The patient was discharged on Day 8 without signs of reperfusion injury or infection. Elderly patients with comorbidities and long-term tissue ischemia may be at risk for reperfusion injury and infection. However, sufficient vascular anastomosis, good tissue quality and optimal evaluation can achieve a good result.

## INTRODUCTION

Replantation is a method of reconnecting tissue or limb followed by the application of microsurgical technique. The first successful scalp replantation was conducted by Miller in 1976 [1]. Reportedly, only a few cases of partial or total scalp amputation can be found in the trauma unit [2, 3].

The most common incidents that led to this trauma are rotating machine accident, animal bite and assault [4, 5]. Scalp amputation can lead to severe injury, permanent hair loss and massive blood loss. Partial defects can be managed by local flaps or skin grafts, while complete detachment may require free flap or replantation, with consideration of ischemia time and blood supplies for scalp survival [1, 6, 7].

Elderly and prolonged ischemia time, followed by a combination of comorbidities, are very susceptible to reperfusion injury or ischemia-induced necrosis. When not properly treated, people who receive suboptimal care will lead to poor clinical outcomes [8].

## CASE REPORT

**Figure 1:**
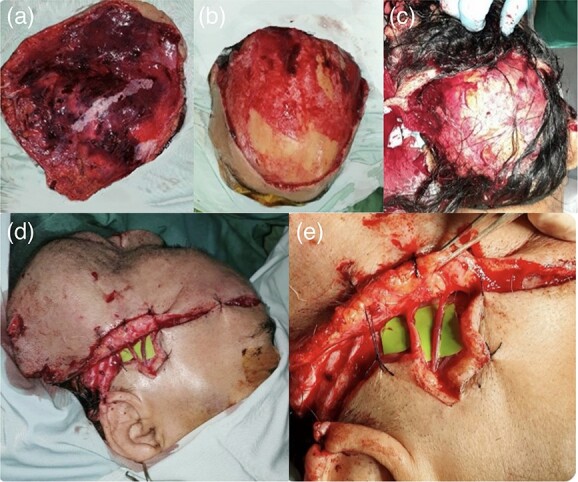
69-year-old woman with traumatic scalp amputation; (**a**) a total circular scalp avulsion with multi-level lacerated wounds; (**b**) an intact skull stump; (**c**) total detachment of the scalp exposing the skull stump; (**d**) scalp attachment with sutures to support the head shape and (**e**) an end-to-end anastomosis of the right superficial temporal artery and vein.

A 69-year-old woman was referred to Mohammad Hoesin Hospital in Palembang, South Sumatra, with total scalp amputation due to dog bites 2 hours before admission. The scalp tissue was 30 × 26 cm in full thickness, with multi-level laceration, irregular edges and exposed skull stump (Fig. 1). The patient also has comorbidities of high glucose index (uncontrolled diabetes mellitus), hypertension and obesity (body mass index > 32), and there are no signs of concussion or airway obstruction.

Hemorrhage control was performed, as well as blood loss resuscitation with fluid and blood transfusion. After obtaining good vital signs, comorbidities were treated preoperatively; this took 9 hours of tissue ischemia time after the incident. Although replantation success may be small due to prolonged tissue ischemia and comorbidities’ time-consuming management, replantation on the scalp was still being carried out.

After removing damaged cells and visible dirt, the scalp was washed several times with normal saline and disinfected with iodine solution. The patient was supine during the operation and under general anesthesia. Because attempts to identify the supratrochlear artery were futile, vessels in the temporal lobe were selected for the anastomosis. Prolene 9.0 microsurgery under a dissecting microscope was used to perform end-to-end anastomosis of the right superficial temporal artery and vein. After 7 hours of anastomosis, the scalp was sutured to the periosteum with prolene 4.0–5.0, and heparin lavage was immediately performed. Nerve anastomosis cannot be performed due to patient morbidity from long hours surgery. The amputated scalp had undergone a total of 18 hours of warm ischemia time.

**Figure 2:**
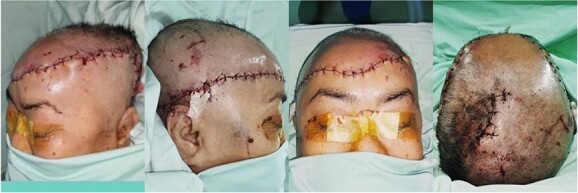
Post-operative.

Post-operatively (Fig. 2), the scalp showed a positive pin-prick test, restored capillary refill time and adequate blood flow as measured by doppler ultrasonography. Every 12 hours, antibiotics and 10 000 IU of heparin were given intravenously. The patient was then admitted to the ICU in an intubated state with close monitoring and was able to be discharged in a stable condition 8 days after surgery.

After 17 days of surgery, several areas along the line of scalp avulsion and multi-laceration wounds were found to be moderately necrotic. The patient was referred for debridement, and honey dressing was applied. Fresh granulation tissue appeared a month later, indicating a successful wound healing.

**Figure 3:**
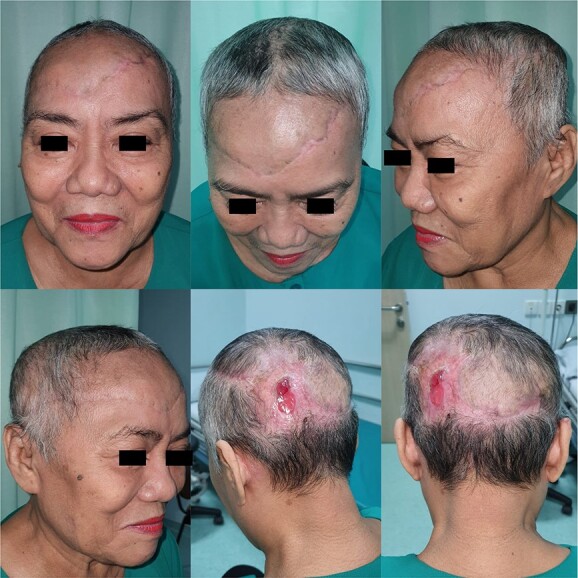
2-month follow-up.

The wound healed well after 2 months, and hair began to grow (Fig. 3). The patient did not require any additional revisions.

**Figure 4:**
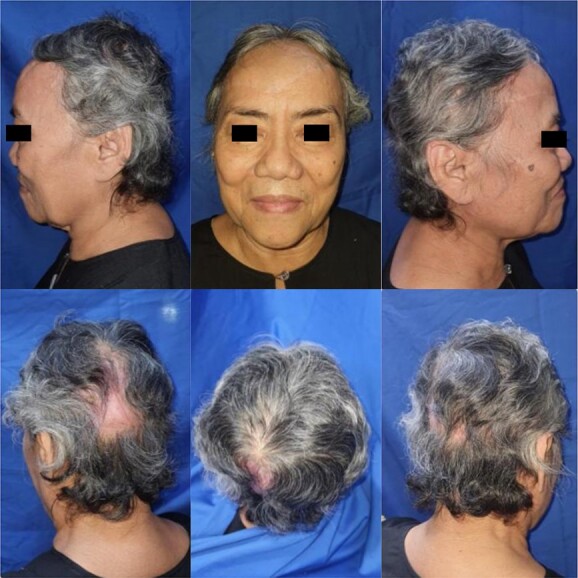
9-month follow-up.

During 9 months follow-up (Fig. 4), the patient regained consequent hair growth, and small alopecia area can be covered through hair styling.

**Figure 5:**
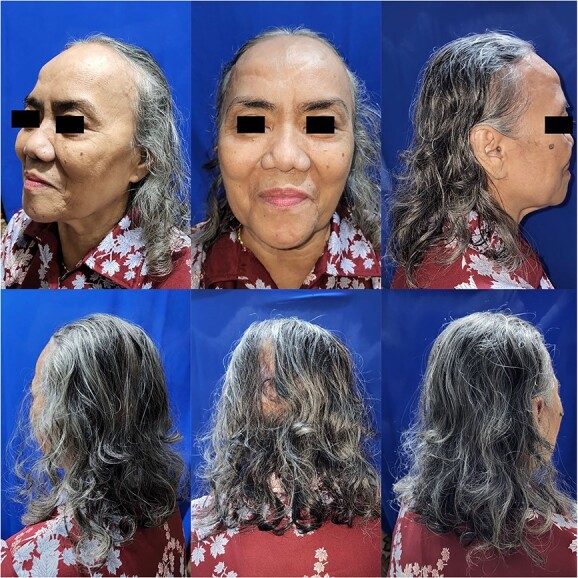
2-year follow-up.

After 2 years (Fig. 5), the patient has no complaints except for paresthesia in several areas of the scalp. However, the patient was very pleased with the outcome.

## DISCUSSION

This replantation of total scalp amputation on an elderly patient has a high risk of failure due to 18 hours of warm ischemia time, patient’s comorbidities and reperfusion injury. Skin and subcutaneous tissue, which are relatively resistant to ischemia, can optimally survive up to 6 hours or more [6]. However, this case of prolonged tissue ischemia has shown an effective result of scalp survival from replantation even for the hair that is least ischemia resistant.

In cases of prolonged ischemia time, predicting whether the scalp can survive is still a debate among reconstructive surgeons. Therefore, scalp survival can be predicted from the quality of the scalp and severity of scalp inner vasculature injury. After surgery, scalp necrosis might occur caused by arterial injury, hence good comprehensive management should be ensured [6].

The presence of comorbidities must be addressed both before and after surgery, as they may be at risk of complications and mortality [8]. Antibiotics are also important for contaminated wounds, particularly for the bacteria most commonly found in dog mouths (*Pasteurella multocida*) [4, 6, 9].

Replantation surgery in the elderly requires an adapted and individualized approach as well as a combination of microsurgery advances. Apart from replantation, other options for total scalp amputation are the Antero-lateral Thigh (ALT) Free flap, which may require up to two thighs due to the large defect, as well as Split-thickness Skin Graft (STSG) after previously chipping the skull and awaiting granulation, which is time-consuming and carries a risk of osteomyelitis.

This case concludes that replantation is the best option for total scalp amputation requiring successful anastomosis, good management of comorbidities and long-term evaluation in order to achieve optimal reconstruction and good esthetic outcome.

## STUDY DESIGN

Retrospective study.
